# Effect of amlodipine and telmisartan on bone metabolism markers in newly diagnosed hypertensive patients

**DOI:** 10.6026/973206300223768

**Published:** 2026-06-30

**Authors:** Kamlesh Garg, Perumal Raveendranath, Sameer Gulati, Sufian Zaheer, Amita Yadav, Ruchika Nandha

**Affiliations:** 1 Department of Pathology, Pharmacology, & Biochemistry, Vardhman Mahavir Medical College & Safdarjung Hospital, New Delhi, India; 2 Department of Medicine, Lady Hardinge Medical College, New Delhi, India; 3 Department of Pharmacology, Dr Harvansh Singh Judge Institute of Dental Sciences & Hospital, Panjab University, Chandigarh, India

**Keywords:** Bone metabolism markers, bone turnover markers, amlodipine, telmisartan, hypertension, parathormone, osteocalcin, interleukin-6, bone-specific alkaline phosphatase

## Abstract

Hypertension may adversely affect bone health, but the skeletal effects of commonly prescribed antihypertensive drugs remain inadequately u nderstood. In this 24-week prospective study, 96 newly diagnosed Stage I hypertensive patients received either Amlodipine (5-10 mg) or Telmisartan (40-80 mg), with serial assessment of bone metabolism markers. Both drugs achieved comparable blood pressure control throughout the study period. Telmisartan significantly reduced PTH and IL-6 levels while enhancing BS-ALP and transiently increasing osteocalcin, whereas Amlodipine demonstrated only a temporary rise in BS-ALP. Thus, data shows the telmisartan may provide additional benefits on bone remodeling and skeletal health in hypertensive patients at risk of fragility.

## Background:

Hypertension is a major global health concern and affects a substantial proportion of the Indian population, particularly adults over 40 years of age [1]. In addition to its established association with cardiovascular and renal disorders, hypertension has emerged as an important risk factor for impaired bone health and osteoporotic fractures. Epidemiological studies have demonstrated a significant association between elevated blood pressure and reduced bone mineral density (BMD), especially among older adults [2]. The relationship between hypertension and skeletal fragility is multifactorial and involves disturbances in calcium homeostasis, chronic inflammation, sympathetic nervous system activation and overactivity of the renin-angiotensin-aldosterone system (RAAS) [3, 4]. Bone metabolism is a dynamic process regulated by the balance between bone formation and bone resorption. Bone turnover markers provide valuable insight into ongoing skeletal remodeling and may detect metabolic changes earlier than radiographic methods. Important bone formation markers include Bone-Specific Alkaline Phosphatase (BS-ALP) and osteocalcin, whereas inflammatory mediators such as Interleukin-6 (IL-6) and hormones including Parathormone (PTH) also play crucial roles in regulating bone turnover [5]. The impact of antihypertensive medications on bone metabolism has gained increasing attention because hypertension and osteoporosis frequently coexist in elderly individuals. Among antihypertensive agents, angiotensin receptor blockers (ARBs) and thiazide diuretics have been reported to exert favorable effects on bone, whereas calcium channel blockers are generally considered bone-neutral [6]. However, evidence regarding their influence on specific bone metabolism markers remains inconsistent. Amlodipine, a widely prescribed dihydropyridine calcium channel blocker, has shown variable effects on skeletal metabolism. Experimental studies suggest that calcium channel blockade may influence osteoblastic activity and fracture healing, but clinical studies have reported inconclusive findings regarding its effects on bone turnover markers and bone mineral density [7]. Telmisartan, an angiotensin II receptor blocker with partial PPAR-γ agonistic activity, may exert additional pleiotropic effects by modulating inflammatory pathways and reducing PTH-mediated bone resorption. Nevertheless, available human studies have produced conflicting results regarding its impact on bone metabolism [8]. Therefore, it is of interest to evaluate the effects of Amlodipine and Telmisartan on bone metabolism markers in newly diagnosed hypertensive patients, with the goal of identifying pleiotropic skeletal benefits relevant to holistic hypertensive management.

## Materials and Methods:

An observational, open-label, prospective study was conducted over 18 months in the Department of Pharmacology, in collaboration with the Departments of Medicine, Pathology and Biochemistry, at Vardhman Mahavir Medical College & Safdarjung Hospital, New Delhi - 110029, after approval from the Institutional Ethics Committee (IEC/VMMC/SJH/Thesis/2020-11/CC-259). Patients attending the Medicine Clinic of VMMC, New Delhi, who were newly diagnosed with Stage I hypertension, fulfilled the inclusion/exclusion criteria and provided informed consent were enrolled. Blood samples were collected after overnight fasting for estimation of serum Calcium, Phosphate, Vitamin D3, PTH, Osteocalcin, IL-6 and BS-ALP at baseline, 12 weeks and 24 weeks. The starting dose for Amlodipine was 5-10 mg once daily at bedtime; for Telmisartan it was 40-80 mg at bedtime. Doses were titrated by the treating physician in cases of inadequate response at one month. Data were entered in Microsoft Excel and analysed using SPSS version 25.0 (IBM Corp., Armonk, NY). Quantitative data are expressed as mean ± SD and qualitative data as proportions or percentages. Chi-square test was used for qualitative variables; paired t-test for intragroup comparisons; unpaired t-test for intergroup comparisons. P-value < 0.05 was considered statistically significant.

## Inclusion criteria:

Newly diagnosed Stage I hypertensive patients above 40 years of age of either gender (SBP 140-159 mmHg and/or DBP 90-99 mmHg according to JNC VIII guidelines) prescribed either Telmisartan or Amlodipine to achieve blood pressure control.

## Exclusion criteria:

Known hypertensive patients already on antihypertensive therapy; patients with comorbid conditions (diabetes, thyroid dysfunction); conditions affecting calcium and bone homeostasis (malabsorption syndrome, Cushing syndrome, parathyroid dysfunction, known malignancy, renal or hepatic disease); patients on drugs affecting calcium and bone homeostasis (calcium/vitamin D supplements, bisphosphonates, corticosteroids, levothyroxine, SERMs); patients with vitamin D deficiency (Vitamin D < 20 ng/ml); and patients requiring combination antihypertensive therapy.

## Results:

A total of 96 newly diagnosed Stage I hypertensive patients were recruited and divided equally into Group A (Amlodipine, n = 48) and Group B (Telmisartan, n = 48). Baseline demographic and clinical characteristics were comparable between groups (p > 0.05) (Table 1). Most participants were in their fifth and sixth decades of life; 53% were male and 47% were female. Both treatments significantly reduced systolic and diastolic blood pressure from baseline. Mean SBP decreased from 150.69 ± 5.5 mmHg to 123.27 ± 4.19 mmHg at 24 weeks in the Amlodipine group and from 150.65 ± 6.0 mmHg to 121.8 ± 2.9 mmHg in the Telmisartan group (within-group p < 0.0001 for both). No significant between-group differences were observed (p > 0.05) (Table 2). Mean Vitamin D3 levels were 23.96 ± 4.08 ng/ml (Amlodipine) and 24.59 ± 4.13 ng/ml (Telmisartan) at baseline. Changes were not statistically significant at 12 or 24 weeks within or between groups (p > 0.05) (Table 3). Mean PTH levels were 53.01 ± 9.98 pg/ml and 54.83 ± 9.74 pg/ml at baseline. Amlodipine did not significantly alter PTH. Telmisartan produced a significant reduction at 24 weeks compared to baseline (p = 0.021). Between-group comparisons were not significant (Table 3, Figure 1 - see PDF). Serum calcium and phosphate remained stable throughout the study in both groups, with no significant intra- or inter-group differences at 12 or 24 weeks (p > 0.05) (Table 3). Mean baseline IL-6 levels were 16.80 ± 8.62 pg/ml and 16.20 ± 7.77 pg/ml respectively. Amlodipine showed no significant effect. Telmisartan significantly reduced IL-6 at 24 weeks (p = 0.002) and between 12 and 24 weeks (p = 0.001). Between-group differences were not significant (p > 0.05) (Table 3, Figure 1 - see PDF). Baseline osteocalcin was 1063.90 ± 1012.62 pg/ml (Amlodipine) and 1012.10 ± 1251.48 pg/ml (Telmisartan). Amlodipine changes were not significant. Telmisartan showed a significant transient increase at 12 weeks (p = 0.042), not maintained at 24 weeks. Between-group differences were not significant (Table 3, Figure 2 - see PDF). Baseline BS-ALP was 38.71 ± 17.64 µg/l and 38.25 ± 20.66 µg/l respectively. Amlodipine showed a significant increase at 12 weeks (p = 0.018), not maintained at 24 weeks. Telmisartan demonstrated significant increases at both 12 weeks (p = 0.033) and 24 weeks (p = 0.034). Between-group differences were not significant (Table 3, Figure 1 - see PDF).

## Discussion:

In the present study, although patients were not randomised, both groups were comparable in terms of age, gender and BMI (p > 0.05). Both systolic and diastolic blood pressures were effectively controlled according to JNC VIII recommendations, with no significant differences between the groups, ensuring that observed differences in bone markers were attributable to the specific pharmacological actions of the two drugs rather than to differences in blood pressure control. Neither Amlodipine nor Telmisartan significantly altered serum Vitamin D3 levels at 12 or 24 weeks. These findings are consistent with those of Bekki *et al.*, which also reported non-significant changes in Vitamin D following therapy with Amlodipine and Telmisartan respectively [9]. Telmisartan produced a gradual and statistically significant reduction in serum PTH at 24 weeks (p = 0.021). Chandler *et al.* [10] noted a decrease in PTH at 12 weeks, but this effect was no longer significant after adjustment for concurrent changes in 25-hydroxy Vitamin D, suggesting that their finding was largely Vitamin D-mediated. In contrast, the significance achieved at 24 weeks in our study in the absence of significant Vitamin D changes suggests a potential time-dependent, independent pleiotropic effect of Telmisartan on the parathyroid-bone axis. The non-significant decrease in PTH with Amlodipine is consistent with the findings of Anwar *et al.* [11]. The significant reduction in serum IL-6 with Telmisartan at 24 weeks (p = 0.002) highlights its potent anti-inflammatory properties, with implications for vascular inflammation, cardiac remodelling and modulation of osteoclastic activity. This is concordant with the meta-analysis by Lu *et al.* [12] which confirmed that Telmisartan consistently reduces pro-inflammatory cytokines across multiple randomised controlled trials. Studies by Zhou *et al.* [13] demonstrated that blockade of the angiotensin II receptor may reduce PTH-mediated bone resorption, supporting the favorable skeletal effects observed with Telmisartan in the present study. Birocale *et al.* [14] reported that long-term Telmisartan use was associated with reduced bone mineral density in animal models, highlighting the ongoing controversy regarding its skeletal actions. Brito *et al.* [15] showed that Telmisartan attenuated inflammatory mediators and promoted bone protection in periodontal disease models, consistent with the reduction in IL-6 observed in our study. Gomaz *et al.* found no significant effect of Telmisartan on osteocalcin levels, whereas our study demonstrated a transient increase at 12 weeks. In contrast, our 24-week findings showed sustained IL-6 reduction and increased BS-ALP without changes in Vitamin D status, indicating a possible direct pleiotropic effect of Telmisartan on bone formation and remodeling. Experimental studies have also demonstrated that Amlodipine can enhance alkaline phosphatase activity in osteoblastic cells, supporting the transient rise in BS-ALP observed in the present study [16].

## Conclusion:

Telmisartan demonstrated sustained favorable effects on bone formation and inflammatory markers independent of serum calcium, phosphate and Vitamin D3 status. In contrast, Amlodipine showed minimal influence on skeletal metabolism over 24 weeks. Thus, Telmisartan may provide added skeletal benefits beyond blood pressure control and could be advantageous in hypertensive patients at risk of bone fragility.

## Ethical Approval:

Approved by Institutional Ethics Committee, VMMC & Safdarjung Hospital (IEC/VMMC/SJH/Thesis/2020-11/CC-259).

## Funding:

No external funding was received for this study.

## Figures and Tables

**Table 1:** Demographic and baseline clinical characteristics

**Parameter**	**Amlodipine (n=48)**	**Telmisartan (n=48)**	**P-value**
Age (years)	48.56 ± 7.96	49.81 ± 7.31	0.425
Gender (M/F)	25/23	26/22	0.838
BMI (kg/m^2^)	25.52 ± 3.14	25.03 ± 2.94	0.426
Baseline SBP (mmHg)	150.69 ± 5.5	150.65 ± 6.0	0.972
Baseline DBP (mmHg)	95.10 ± 2.76	94.83 ± 2.03	0.586

**Table 2:** Blood pressure comparison at baseline, 12 Weeks and 24 Weeks

	**SBP (mmHg)**			**DBP (mmHg)**		
	Baseline	12 Weeks	24 Weeks	Baseline	12 Weeks	24 Weeks
Amlodipine Group	150.69±5.5	127.25±4.9	123.27±4.19	95.10±2.76	85.54±2.02	84.15±2.00
Telmisartan Group	150.65±6.0	128.52±5.12	121.8±2.9	94.83±2.03	85.04±2.40	83.25±2.14

**Table 3:** Comparison of bone metabolism markers over 24 weeks (significant p-values in bold red)

**Marker**	**Group**	**Baseline**	**12 Weeks**	**24 Weeks**	**p [BL vs 12w]**	**p [BL vs 24w]**	**p [12w vs 24w]**
Vit D3 (ng/ml)	Amlodipine	23.96±4.08	24.67±3.24	24.73±3.43	0.154	0.147	0.863
	Telmisartan	24.59±4.13	25.19±3.73	25.41±3.37	0.206	0.193	0.668
	P (between groups)	0.459	0.466	0.332			
PTH (pg/ml)	Amlodipine	53.01±9.98	53.71±10.59	50.29±11.48	0.707	0.145	0.18
	Telmisartan	54.83±9.74	52.63±10.61	50.73±13.42	0.301	0.021	0.439
	P (between groups)	0.369	0.62	0.862			
Calcium (mg/dl)	Amlodipine	9.30±0.39	9.33±0.37	9.27±0.31	0.469	0.65	0.157
	Telmisartan	9.24±0.36	9.24±0.34	9.24±0.32	0.926	0.858	0.9
	P (between groups)	0.417	0.208	0.651			
Phosphate (mg/dl)	Amlodipine	4.16±0.46	4.17±0.34	4.20±0.34	0.409	0.189	0.312
	Telmisartan	4.11±0.37	4.17±0.40	4.17±0.29	0.133	0.211	0.957
	P (between groups)	0.783	0.978	0.58			
IL-6 (pg/ml)	Amlodipine	16.80±8.62	16.45±8.39	15.86±7.84	0.355	0.052	0.183
	Telmisartan	16.20±7.77	15.61±6.77	13.79±5.64	0.162	0.002	0.001
	P (between groups)	0.721	0.593	0.141			
Osteocalcin (pg/ml)	Amlodipine	1063.90±1251.62	1331.62±1149.79	1169.77±825.19	0.149	0.582	0.395
	Telmisartan	1012.10±1251.48	1493.25±1577.37	1253.75±1530.58	0.042	0.256	0.224
	P (between groups)	0.824	0.568	0.739			
BS-ALP (µg/l)	Amlodipine	38.71±17.64	51.69±33.18	46.02±51.69	0.018	0.319	0.53
	Telmisartan	38.25±20.66	65.08±78.71	52.85±40.21	0.033	0.034	0.075
	P (between groups)	0.907	0.28	0.471			
BL = Baseline.
Bold red values
indicate p <
0.05 (statistically significant).
